# Application of Biomass-Based Triboelectrification for Particulate Matter Removal

**DOI:** 10.3390/polym16202933

**Published:** 2024-10-18

**Authors:** Hui Chen, Yabo Wu, Zheng Ma, Yefei Wu, Zhaodong Ding, Lianghong Yin

**Affiliations:** 1 State Key Laboratory of Subtropical Silviculture, Zhejiang A&F University, Hangzhou 311300, China; huichen9411@163.com (H.C.); 13777331213@163.com (Y.W.); 2 Zhejiang Provincial Key Laboratory of Resources Protection and Innovation of Traditional Chinese Medicine, Zhejiang A&F University, Hangzhou 311300, China; 3 Valmet Paper Technology (China) Co., Ltd., Wuxi Service Center, Wuxi 214028, China; 4 Zhejiang Provincial Key Laboratory of Biometrology and Inspection & Quarantine, College of Life Sciences, China Jiliang University, Hangzhou 310018, China; mazheng520@163.com; 5 Zhejiang Qianjiang Biochemical Co., Ltd., Haining 314400, China; yefei0906@163.com

**Keywords:** triboelectric nanogenerator, cellulose aerogel, airborne pollutants, Ti_3_C_2_T_x_, facemask

## Abstract

Electrostatic fields are crucial for achieving the highly efficient filtration of airborne pollutants. However, the dissipation of static charges over time, especially under humid conditions, limits their practical application. In this study, we present a self-charging air filter (SAF) powered by a triboelectric nanogenerator (TENG). This SAF is integrated into a commercial mask, termed SAFM, which can effectively capture and degrade airborne pollutants without requiring an external power source. By leveraging the triboelectric effect during breathing, the TENG within the SAFM continuously replenishes static charges, maintaining the triboelectric field. The system employs a cellulose aerogel/Ti_3_C_2_T_x_ composite as the electron donor and an esterified cellulose-based electrospun nanofiber as the electron acceptor. Remarkably, the triboelectric field significantly enhances filtration performance, with the SAF achieving up to 95.7% filtration efficiency for particulate matter as small as 0.3 μm. This work underscores the potential of TENG-powered triboelectric fields in the development of multifunctional, human-machine interactive facemasks.

## 1. Introduction

Influenza viruses are primarily transmitted through airborne particles exhaled by an infected person during activities like coughing, sneezing, speaking, singing, or even breathing [1,2,3]. These viruses can cause serious complications, such as pneumonia or cardiopulmonary failure, particularly in elderly individuals, children with weakened immune systems, and patients with immunodeficiency disorders. Symptoms like dyspnea, shortness of breath, and irregular breathing patterns have made respiratory monitoring a critical tool in preventive diagnostics, significantly reducing infection and mortality rates associated with respiratory diseases [4]. As breathing is a vital physiological process involving oxygen intake and carbon dioxide exhalation, the development of an intelligent, wearable facemask capable of intercepting virus transmission is of paramount importance [5,6]. This highlights the need for more efficient and durable filtration solutions.

Commercial surgical masks consist of three layers: a polypropylene melt-blown fabric as the filtration medium, flanked by two layers of polyethylene nonwoven fabric. The inner layer is hydrophilic to absorb exhaled moisture, while the outer layer is hydrophobic to repel fluids [7]. However, these masks rely on mechanical filtration mechanisms (inertial impaction, interception, sieving, and diffusion) and need replacement every 2–4 h due to a reduction in their efficiency in filtering particulate matter (PM) [8,9].

To address these limitations, there is an urgent need for a portable, self-sustaining facemask capable of a higher filtration efficiency and extended wearability. While electrospinning technology offers improved mechanical filtration over traditional melt-blown fabrics due to the presence of finer fibers, small particulate matter (PM) can still evade capture [10,11]. To address this, functional materials like Ti_3_C_2_T_x_, a two-dimensional (2D) transition metal carbide, have been explored for their unique conductivity and pollutant adsorption properties, which can enhance mask performance [12,13,14,15]. In addition to material improvements, electrostatic fields play a key role in enhancing filtration by capturing ultrafine particles through electrostatic adsorption [16]. Through corona electret charging, ultrafine PM can be captured by injecting an electrostatic charge into the filter media without causing a significant pressure drop [17]. Electrostatic adsorption can account for up to 80% of the overall filtration efficiency [18,19]. However, the gradual dissipation of charge reduces filtration efficiency over time, limiting the effectiveness of traditional electrostatic filters [20,21,22].

To overcome these challenges, triboelectric nanogenerators (TENGs) offer a promising solution by continuously generating electrostatic charge from ambient mechanical energy, such as human motion or breathing [23,24,25,26,27,28]. TENG-powered facemasks provide a self-sustaining electrostatic field, enhancing filtration efficiency without requiring external power [29,30]. Recent studies have demonstrated the effectiveness of TENGs in capturing PM and enabling respiratory monitoring [5,19,26,31]. Thus, integrating TENG-generated electrostatic fields into commercial facemasks offers significant benefits, improving air pollutant filtration efficiency while enabling continuous monitoring of key respiratory symptoms.

In this study, building on our previous research [32], we developed a self-charging air filter (SAF) powered by a TENG and incorporated it into a commercial facemask (referred to as SAFM) to achieve high-efficiency PM filtration without the need for external power. The TENG-based SAF continuously generates charge and maintains a triboelectric field through triboelectrification, utilizing a composite of cellulose aerogel (CA)/Ti_3_C_2_T_x_ as the electron donor and an esterified electrospun polyvinyl alcohol (PVA)–cellulose nanofiber (CNF)–polyethylene oxide (PEO) film as the electron receiver during respiration. With the support of this triboelectric field, the SAF achieves a filtration efficiency of up to 95.7% for 0.3 μm PM, significantly enhancing overall filtration performance. This study broadens the application of TENG-based triboelectric fields for airborne pollutant removal, showcasing their significant potential for multifunctional intelligent facemasks.

## 2. Materials and Methods

### 2.1. Materials

Polyvinyl alcohol (PVA, M_W_ = 17,000 ± 50), 3% TEMPO-oxidized cellulose nanofibers (CNF), and polyethylene oxide (PEO) were purchased from Sigma-Aldrich (St. Louis, MO, USA). Lithium fluoride (LiF), titanium aluminum carbide (Ti_3_AlC_2_), citric acid (CCA), phosphoric acid (H_3_PO_4_), and all other chemicals used in this study were of analytical grade and were also obtained from Sigma-Aldrich.

### 2.2. Ti_3_C_2_T_x_ Synthesis

Ti_3_C_2_T_x_, the first MXene 2D compound reported in 2011, was followed by the discovery of approximately 30 additional compositions, all with the general formula M_n+1_X_n_T_x_. In this formula, M represents an early transition metal (such as Ti, Zr, Hf, V, Sc, Y, Nb, Ta, or Mo), X denotes carbon and/or nitrogen, and T_x_ refers to surface-terminating functional groups like -OH, -F, -O, and -Cl [33]. Ti_3_C_2_T_x_ has been the most extensively studied due to its remarkable properties, including high metallic conductivity and chemical stability. In this study, we employed a mild etching method to synthesize Ti_3_C_2_T_x_ [34]. First, LiF (0.5 g) was completely dissolved in 9 M HCl (5 mL) and vigorously stirred at 800 rpm for a duration of 60 min. Subsequently, Ti_3_AlC_2_ was gradually introduced into the resulting solution. After 24 h of stirring, the resulting product was subjected to centrifugation and washed with deionized water for 10 cycles until the pH of the supernatant reached 7, leading to the separation of the precipitate. Finally, the obtained product was lyophilized to obtain a multi-layer Ti_3_C_2_T_x_ powder.

### 2.3. Preparation of CNF-Based Aerogel (CA)

PVA (0.3 g) was dissolved in 100 mL of a Tempo-oxide CNF suspension (0.5 wt%) under magnetic stirring at 90 °C for 2 h, followed by cooling to room temperature. Subsequently, a mixture of citric acid (CCA) (1.2 g) and phosphoric acid (H_3_PO_4_) (1.2 mL) was added to the suspension with continuous stirring for 1 h. The resulting suspensions were transferred into a plastic beaker and rapidly frozen in liquid nitrogen for 5 min. Following complete freezing, the samples were subjected to freeze-drying in a freezer dryer to obtain aerogels, which then underwent crosslinking between PVA and CCA by heating at 60 °C for 2 h in an oven. The samples were subsequently subjected to thorough washing with deionized water and then dried at ambient temperature.

### 2.4. Preparation of PVA-CNF-PEO Film

An electrospun film was produced using a suspension comprising PVA, 3% CNF (based on the dry weight of PVA), and 8% PEO (the total dry weight of the PVA and CNF was used as the reference). An aqueous suspension was loaded into a 10 mL syringe to produce a fluid jet under ambient conditions, with a relative humidity of 45% and a temperature of 25 °C. The procedure was carried out with a 30 kV voltage applied, maintaining a flow rate of 2 mL/h for a duration of 6 h.

### 2.5. Assembly of the Self-Charging Mask

The CA/Ti_3_C_2_T_x_ and PVA-CNF-PEO materials were shaped into 2 cm diameter circles, acting as the positive and negative triboelectric layers. Two copper foil electrodes, each 0.1 mm thick and ring-shaped, were attached around the edges of the CA/Ti_3_C_2_T_x_ and PVA-CNF-PEO layers, respectively. The ring-shaped support layer made of polylactic acid with a thickness of 2 mm was utilized to maintain the gap between CA/Ti_3_C_2_T_x_ and PVA-CNF-PEO. Two wires were connected to CA/Ti_3_C_2_T_x_ and PVA-CNF-PEO, facilitating current flow to the external circuit. The SAF integration into a commercial mask shell resulted in the construction of a self-charging mask known as SAFM.

### 2.6. Filtration Performance Testing Platform

Filtration performance was evaluated using a dedicated testing setup. PM was produced through incense burning and was introduced via compressed air, with the flow rate adjusted to regulate the PM concentration. A PM counter was employed to determine the concentration in 1 L of air, and a differential pressure gauge was used to track the pressure drop across the sample. Once the sample was in place, a thermal anemometer was utilized to accurately measure and log the air flow rate.

### 2.7. Characterization

The surface morphology and elemental compositions of CA, Ti_3_C_2_T_x_ powder, and CA/Ti_3_C_2_T_x_ were examined using a scanning electron microscope (SEM) (Jeol Neoscope JCM-5000, Jeol, Tokyo, Japan). These samples were freeze-dried and then fractured in liquid nitrogen to preserve the micromorphology. The fracture surfaces were sputter-coated with gold for 80 s before SEM examination, while the crystal structure was determined via XRD using a Smartlab-3KW + UltimaIV3KW instrument (Rigaku Corporation, Tokyo, Japan) with a scanning speed of 5°/min and a range of 5°–30°. Contact-angle measurements were performed using a Ramé-Hart model 500-F1 system (Ramé-Hart Instrument Co., Succasunna, NJ, USA). The electrical output performance of the SAF was tested with a Keithley 6514 electrometer (Tektronix Inc., Beaverton, OR, USA), while a digital linear motor (LinMot H01-23386/160, NTI AG LinMot, Spreitenbach, Switzerland) was utilized to carry out the contact and separation cycles under different forces and frequencies.

## 3. Results and Discussion

### 3.1. Preparation and Characterization of CA/Ti_3_C_2_T_x_ Composites

**Figure 1:**
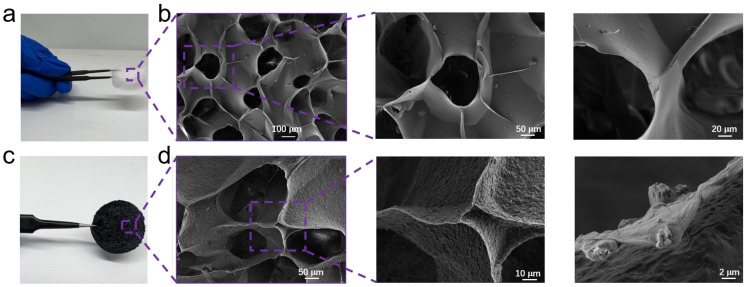
(**a**) Photograph and (**b**) SEM image of CA. (**c**) Photograph and (**d**) SEM image of CA/Ti_3_C_2_T_x_.

As described in Section 2.3, a robust, cross-linked, three-dimensional CA framework with a density of 10.7–12.4 mg/cm^3^ was successfully produced (Figure 1a). The CA film was then immersed in a 0.5 wt% Ti_3_C_2_T_x_ solution (100 mL), followed by sonication for 60 min and stirring at 25 °C for 1 h. This process facilitated the uniform in situ growth of Ti_3_C_2_T_x_ on the surface of the CA, resulting in the formation of a CA/Ti_3_C_2_T_x_ composite. Finally, the dried films were cured at 100 °C. The three-dimensional honeycomb network of the CA displayed a well-ordered network structure with a uniform pore size distribution and thin pore walls. The surface appeared to be smooth, and a micro-scale pore structure could be observed, which is beneficial for applications in air filtration and adsorption. The magnified images further reveal the detailed microstructure of the CA (Figure 1b).

Ti_3_C_2_T_x_ is an attractive type of highly porous solid with potential applications in capturing airborne PM [35]. Despite Ti_3_C_2_T_x_ incorporation, the overall morphology of the CA/Ti_3_C_2_T_x_ remained unchanged (Figure 1c). Figure 1d presents the SEM images of the CA/Ti_3_C_2_T_x_. Compared to CA, the CA/Ti_3_C_2_T_x_ still maintains a three-dimensional honeycomb structure, but the surface is rougher, and small particles are visible on the pore walls, indicating the uniform attachment of the polyhedral Ti_3_C_2_T_x_ particles to the surface of the CA pore wall. The magnified images clearly show the deposition of CA/Ti_3_C_2_T_x_ on the pore walls, further confirming the composite nature of the material [36].

**Figure 2:**
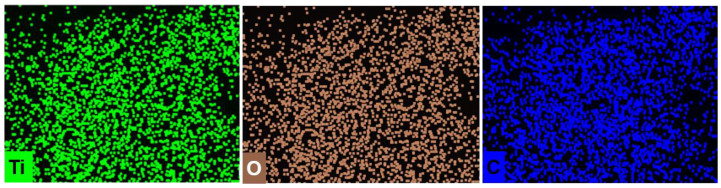
Elemental mapping images of CA/Ti_3_C_2_T_x_ for Ti, O, and C.

**Figure 3:**
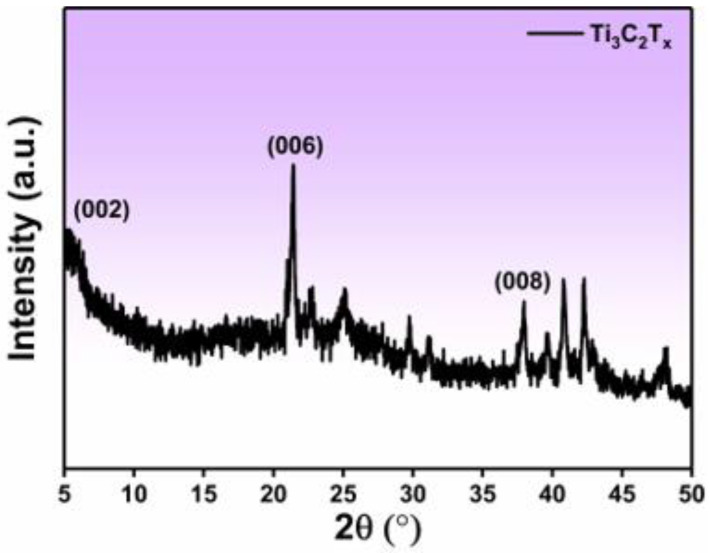
XRD patterns of CA/Ti_3_C_2_T_x_.

Compared to CA, the uniform distribution of the Ti in CA/Ti_3_C_2_T_x_ composite, as evidenced by elemental mapping, further confirmed the successful in situ loading of Ti_3_C_2_T_x_ onto the CA surface (Figure 2). The well-distributed Ti, oxygen, and carbon elements indicate an even dispersion of Ti_3_C_2_T_x_ particles across the CA matrix. This homogeneous distribution plays a crucial role in maintaining the composite’s structural integrity and functional properties. Additionally, XRD analysis further supports the successful preparation of the CA/Ti_3_C_2_T_x_ composite. The appearance of characteristic diffraction peaks at 5.1°, 11.8°, 21.7°, and 37.9° (Figure 3) corresponds to the (002), (006), and (008) planes of Ti_3_C_2_T_x_. The (002) peak at 5.1° suggests the preservation of the layered structure of Ti_3_C_2_T_x_, while the additional peaks confirm the presence of Ti_3_C_2_T_x_ in the composite. The absence of other significant peaks indicates that the CA matrix does not interfere with the crystal structure of Ti_3_C_2_T_x_ [37].

### 3.2. Structure and Filtration Principle of SAFs

**Figure 4:**
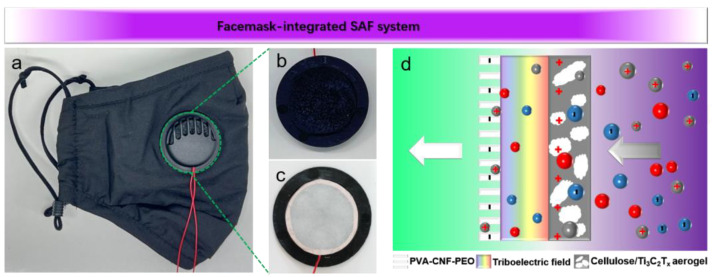
(**a**) Photograph of the facemask assembled with an SAF. (**b**) Photograph of the CA/Ti_3_C_2_T_x_ composite. (**c**) Photograph of an electrospun PVA-CNF-PEO. (**d**) Schematic illustrating the adsorption of a triboelectric field.

Maintaining charges on the filter surface is a critical factor in ensuring the efficient adsorption of electrostatic fields. Electrostatic adsorption is reported to contribute up to 80% of the total filtration efficiency, indicating that electrostatic charge decay poses a limitation on the mask’s lifespan [19,38,39,40]. To address this, we developed an SAF capable of continuously replenishing electrostatic charges, which was then incorporated into an SAFM (Figure 4a–c). The TENG-structured SAF consists of an electrospun PVA-CNF-PEO film and CA/Ti_3_C_2_T_x_ composites in which the PVA-CNF-PEO layer serves as an inwardly worn hydrophobic and electron-accepting filter, while the CA/Ti_3_C_2_T_x_ layer, worn outwardly, acts as an electron donor.

**Figure 5:**
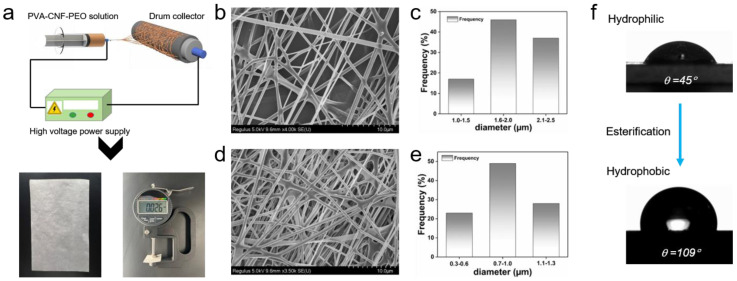
(**a**) Schematic of fabricating PVA-CNF-PEO film by electrospinning. (**b**) SEM image and (**c**) diameters of commercial melt-blown polypropylene film. (**d**) SEM image and (**e**) diameters of electrospun PVA-CNF-PEO film. (**f**) Contact angle of PVA-CNF-PEO and esterified PVA-CNF-PEO at 180 s.

We utilized and characterized the PVA-CNF-PEO film prepared in our previous study [32]. Figure 5a illustrates the preparation procedure, physical appearance, and thickness of the electrospun PVA-CNF-PEO film, which was achieved through electrospinning. The PVA-CNF-PEO filter medium consists of a dense network of randomly crosslinked nanofibers, as observed in the SEM image (Figure 5d). The average fiber diameter was measured to be 847 nm ± 319 nm, with a distribution skewed toward smaller diameters (Figure 5e), contributing to the creation of a three-dimensional, micro-nanoscale porous structure. These pores are formed by multilayer, stacked, nanofiber networks, which significantly enhance the material’s filtration efficiency. Compared to the polypropylene (PP) microfibers commonly used in commercial surgical masks (Figure 5b,c), the nanoscale fibers in the electrospun PVA-CNF-PEO filter medium offer a superior mechanical filtration performance. This is attributed to the smaller fiber diameter and higher surface area, which enable the capture of smaller airborne particles more effectively. In addition, the esterification of the PVA-CNF-PEO nanofibers altered their surface properties, increasing the hydrophobicity of the material. The water contact angle increased significantly from 45° for the untreated PVA-CNF-PEO film to 109° for the esterified version (Figure 5f). This enhancement in hydrophobicity is crucial for preventing charge loss in the filtration medium due to moisture infiltration from exhaled breath, ensuring the sustained performance of the material in humid environments.

Under the driving force of respiration, CA/Ti_3_C_2_T_x_ and PVA-CNF-PEO with varying polarities underwent periodic contact separation to induce a triboelectric field. The negatively charged PMs were drawn into the SAF, where they encountered the positively charged CA/Ti_3_C_2_T_x_ on the outer layer and subsequently underwent adsorption (Figure 4d). Meanwhile, uncharged PMs became polarized after passing through the triboelectric field and were subsequently repelled by the negatively charged PVA-CNF-PEO on the inner layer. The triboelectric field effectively facilitated the interception of PMs through the effects of polarization, like-charge repulsion, and opposite-charge attraction [41,42].

### 3.3. Working Principle and the Electrical Output of the SAF

**Figure 6:**
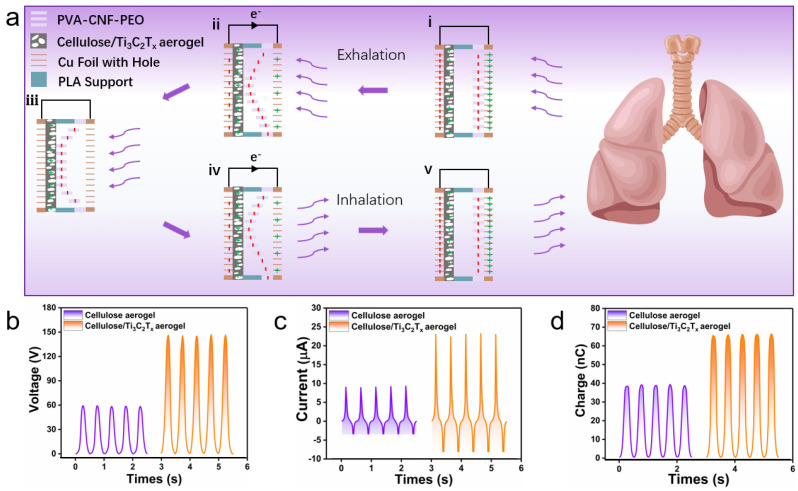
(**a**) Triboelectric charge generation mechanism of the SAF during respiration. (**b**) Output voltage, (**c**) current, and (**d**) transferred charges of the SAF tribomaterial with and without Ti_3_C_2_T_x_.

The process of converting respiration into electricity in SAFs mainly relies on a combination of contact electrification and electrostatic induction. To explain this respiration-to-electricity conversion, an electron cloud model has been introduced. CA/Ti_3_C_2_T_x_ and PVA-CNF-PEO are periodically activated by respiratory airflow, causing them to come into contact and then separate, thus converting the energy from respiration into electrical signals (Figure 6a). In the initial state, the transfer of charges was unobserved between the two non-contacting tribolayers (PVA-CNF-PEO and CA/Ti_3_C_2_T_x_) (Figure 6a(i)). During exhalation, the CA/Ti_3_C_2_T_x_ layer loses electrons due to differing electron affinities, acquiring a positive surface charge, while the PVA-CNF-PEO nanofiber layer gains electrons and becomes negatively charged. This charge transfer shifts the Fermi levels of the Cu electrodes, inducing an electrostatic potential that generates current flow. (Figure 6a(ii)). At the end of the exhalation process, the two tribomaterials make full contact, neutralizing their surface charges and halting the current flow (Figure 6a(iii)). As inhalation begins, the PVA-CNF-PEO layer retracts to its original position, causing the two tribolayers to separate. Trapped surface charges induce electrostatic effects, creating a potential difference between the Cu electrodes, and generating an opposing current flow (Figure 6a(iv)). After the separation of PVA-CNF-PEO from CA/Ti_3_C_2_T_x_ and its restoration to its original state, electron transfer between the electrodes balances the Fermi level difference, resulting in zero external current flow (Figure 6a(v)).

CA and CA/Ti_3_C_2_T_x_ were separately used as positive tribolayers to investigate the effect of Ti_3_C_2_T_x_ on triboelectric properties, under a contact area of 6 cm^2^, frequency of 2 Hz, force of 10 N, and a distance of 2 mm during compression–release cycles. Compared to CA, the open-circuit voltage of CA/Ti_3_C_2_T_x_ increased from 69.7 V to 148.7 V (Figure 6b). This increase in voltage is attributed to the enhanced surface charge density due to the presence of Ti in Ti_3_C_2_T_x_, which improves triboelectric properties. Additionally, the short-circuit current and transferred charge increased from 9.3 to 23.8 μA. The significant rise in current is linked to a higher electron transfer capability, attributed to the Ti_3_C_2_T_x_ additive (Figure 6c). Figure 6d shows the transferred charge as a function of time for the two materials. The charge transfer increased from 38.4 to 63.6 nC, which is associated with a larger surface contact area provided by the in situ loading of Ti_3_C_2_T_x_. The nanostructures introduced on the CA surface by Ti_3_C_2_T_x_ enhanced its contact area with the PVA-CNF-PEO layer [43,44].

### 3.4. Filtration Performance of the SAF

The SAF’s filtration performance was evaluated on a laboratory-designed testing platform. An airflow containing particles of PM_0.3_, PM_2.5_, and PM_10_ was generated by using an air pump in conjunction with smoke produced from burning sandalwood incense. This mixture was then directed into an acrylic box with internal dimensions of 40 cm × 40 cm × 50 cm, where it passed through the test filter film (2.5 cm in diameter). The airflow rate was consistently kept at 12 cm/s, unless specified otherwise.

Notably, the CA/Ti_3_C_2_T_x_ and PVA-CNF-PEO of SAF were initially rubbed together for 20 min to increase surface potential. This is due to the fact that increasing surface charge has been demonstrated to significantly enhance filtration efficiency through electrostatic adsorption while maintaining the air permeability of the filter material [45]. The PM numbers were measured with a PM counter, and the removal efficiency was determined using the following formula [32]:

(1) $$\eta =\frac{c_{0}-c_{1}}{c_{0}}\times 100\%,$$

In this equation, *c*_0_ and *c*_1_ denote the particle concentration of PMs before and after passing through the filter medium, respectively.

**Figure 7:**
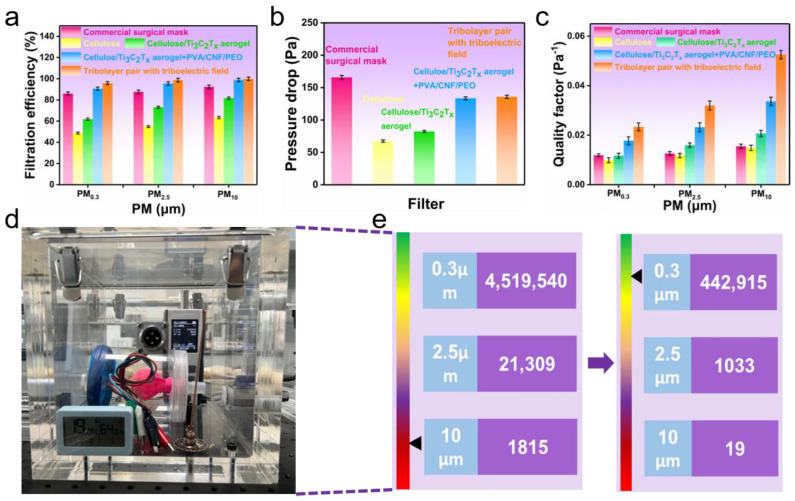
(**a**) Filtration efficiency based on PM diameter. (**b**) Pressure drops corresponding to different filter media. (**c**) Quality factor corresponding to PM diameter. (**d**) Optical photograph showing a simulator of the human lung. (**e**) The PM removal efficiency of the simulator for human lungs.

Figure 7a shows that the overall filtration efficiency increased as the PM size ranged from 0.3 to 10 μm. From this point onward, unless otherwise noted, filtration efficiency refers to 0.3 μm PM, as this size demonstrates the highest penetration and the lowest filtration rate. Specifically, the removal efficiency showed a slight increase compared to commercial surgical masks, CA/Ti_3_C_2_T_x_ + PVA-CNF-PEO, and SAF with the triboelectric field, with values reaching 86.1%, 90.6%, and 95.7%, respectively. For the CA and CA/Ti_3_C_2_T_x_ filter, the efficiency considerably dropped to only 48.7% and 61.5%, respectively. The improvement in filtration efficiency due to Ti_3_C_2_T_x_ was attributed to its large specific surface area and small pore size. The filtration efficiency of CA/Ti_3_C_2_T_x_ + PVA-CNF-PEO exceeded that of CA/Ti_3_C_2_T_x_ by 29.1% as adding a PVA-CNF-PEO film increased the thickness of the filter media, compelling the PMs to travel a longer distance to pass through. The self-powered SAF exhibited a 5.1% higher filtration efficiency than CA/Ti_3_C_2_T_x_ + PVA-CNF-PEO, attributed to the presence of triboelectric field adsorption [46,47].

The increase in removal efficiency is associated with greater breathing resistance (pressure drop), attributed to the larger surface area, which enhances the air’s viscous forces between the filter media and PM (Figure 7b). The quality factor (QF), an essential metric for assessing an air filter’s performance, is determined using the following equation [48]:

(2) $$\mathrm{QF}\;=\frac{-\ln\left(1-\eta \right)}{\Delta p},$$

In this equation, Δ*_p_* refers to the pressure drop measured across the filter during testing.

Although surgical masks achieve a high filtration efficiency, they also exhibit a significant pressure drop (165.8 Pa), leading to a relatively low QF of 11.9 kPa^−1^. Although the CA exhibited a decent pressure drop of 67.6 Pa, its poor efficiency resulted in a low QF of only 9.9 kPa^−1^. The SAF demonstrated an optimal QF of 23.4 kPa^−1^, due to its well-balanced combination of removal efficiency and pressure drop (135.8 Pa), outperforming other filter materials (Figure 7c). Table 1 compares the performance of various air filters based on key parameters such as filtration efficiency, pressure drop, and quality factor, specifically focusing on their ability to filter 0.3 μm particles. Although the performance parameters of SAF are slightly lower than those of some filters powered by TENGs, with high-voltage power supplies or high output voltage, it still demonstrates excellent filtration performance, especially at low airflow pressures. These self-charging filters maintain their charge through a triboelectric field, continuously enhancing the filtration effect and sustaining high filtration efficiency.

To enhance the protection of human health, a simulator of the human lung was utilized to evaluate the SAF’s filtration efficiency in real-world usage scenarios. This experimental setup includes various components such as a flow meter, pressure gauges, and an air sampling interface. This simulator is critical for assessing the performance of air filtration systems, like the SAF, under conditions that closely resemble human breathing patterns (Figure 7d). Under conditions of air pollution, Figure 7e details the PM removal efficiency of the lung simulator when equipped with the SAF. For PM_0.3_, the concentration dramatically drops from 4,519,540/L to 442,915/L, demonstrating a high removal efficiency of 90.2%. Figure 7e also shows similar efficiencies for larger particles (2.5 μm and 10 μm), underlining the SAF’s capability to significantly reduce particle penetration and thereby potentially enhance protection against air pollution.

### 3.5. Economic Evaluation

Durability and cost were assessed using qualitative scoring methods. Durability refers to the stability of electrostatic charges [52,53]. The self-charging method employed in this study can continuously replenish electrostatic charges through mechanical oscillation, such as breathing, without any penalty (five points). Onsite charging involves integrating a micro-button battery into the filter for charge provision on site (one point), but this method loses one point due to potential safety issues and battery replacement requirements. Offsite charging methods have been reported in several studies. An external high-voltage power source, such as a commercial high-voltage power supply or a triboelectric nanogenerator with a high output voltage, is required for charging the filter medium. However, this method incurs a two-point deduction due to the cumbersome and non-portable nature of the high-voltage equipment. Both the N95 respirator (as well as other respirators with comparable levels of protection, such as KN95) and the surgical mask undergo an electret treatment during production. However, these charges gradually dissipate over time, particularly in a humid environment. Consequently, they are awarded two points. Finally, other masks such as cotton masks, medical masks, and activated carbon masks do not undergo an electret treatment process and lack a filter medium altogether, resulting in them receiving only one point. Therefore, the self-charging SAF has the best durability. The comprehensive rating criteria for durability can be found below (Table 2).

The costs for surgical masks, cotton masks, N95 masks, and KN95 masks amount to USD 0.6, USD 0.3, USD 2.5, and USD 1.8, respectively [54,55]. The prices of raw materials utilized in the SAF are documented in Table 3 [56,57]. Owing to the minimal utilization of raw materials, the cost per unit of SAF was as low as 0.0911 USD, making it the most economically viable option for masks. Besides its high filtration efficiency and electrostatic charge stability, SAF also demonstrated favorable respiratory resistance, quality factor, cost-effectiveness, and durability when compared to commercial facemasks.

## 4. Conclusions

The decay of charge in an electret mask undermines its high-efficiency filtration of harmful airborne PMs. This study introduced an efficient, durable, and cost-effective respiration-driven air filter that can continuously replenish electrostatic charges in a self-charging manner. This study first leveraged the triboelectric effect between a CA/Ti_3_C_2_T_x_ (as an electron donor) and esterified PVA-CNF-PEO nanofibers (as electron receivers). TENG-structured SAF could continuously replenish electrostatic charges through breathing. The application of the triboelectric field of TENG substantially enhanced both the PM filtration efficiency. As a result, the filtration efficiency of SAF for 0.3 μm PMs reached 95.7%. In addition, the raw materials for SAF are inexpensive, offering significant economic advantages. The present study considerably broadens the scope of self-powered TENG technology in electrostatic adsorption, showing its excellent potential for multifunctional man-machine interactive masks.

## Figures and Tables

**Table 1:** Source data for the filter performance, pressure drop, and quality factor comparison with previously reported filters.

Filtration Efficiency (%)	Pressure Drop (Pa)	Quality Factor (kPa^−1^)	Challenge Particle Size (μm)	Charging Source	Reference
94	110	25	0.3	TENG with high output voltage	[49]
96	182	25	0.3	High voltage power supply	[50]
99.52	148	44	0.3	TENG with high output voltage	[51]
92.7	86	30.5	0.3	Self-charging with triboelectrification	[19]
95.7	135.8	23.4	0.3	Self-powered with triboelectrification	This work

**Table 2:** The comprehensive rating criteria for durability.

	Self-Charging	Onsite Charging	Offsite Charging	Electret Treatment	Without Charging
Rating	5	4	3	2	1

**Table 3:** Cost calculation for the SAF.

SAF Component	Price (USD)	Usage Per SAF	Cost Per SAF (USD)
PVA	106/Kg	0.085	0.0090
PEO	125/Kg	0.0085	0.0011
CNF	202/Kg	0.00187	0.00038
Stearic acid	88/Kg	0.001	0.000088
CCA	99/Kg	0.0675	0.0067
Ti_3_C_2_T_x_	492/Kg	0.15	0.0738
Total	-	-	0.0911

## Data Availability

The original contributions presented in the study are included in the article, further inquiries can be directed to the corresponding author.
